# The Employment Consequences of Disability After Age 50: Do Working Conditions Matter?

**DOI:** 10.1093/geroni/igaf122.1343

**Published:** 2025-12-31

**Authors:** Constance Beaufils

**Affiliations:** King’s College London, King’s College London, England, United Kingdom

## Abstract

This study examines how employment responses to disability vary with working conditions. We use data from the English Longitudinal Study of Aging and focus on individuals aged 50+ who experience the onset of one or more difficulties with activities of daily living (ADL) or instrumental activities of daily living (IADL) between two waves (N = 992). We combine coarsened exact matching and entropy balancing with logistic modelling to estimate the impact of the onset of disability on the probability of leaving paid work, working part-time, changing jobs or looking for a new job. We examine the moderating role of gender and working conditions (job autonomy, job demands, job physicality). Disability significantly increases the probability of leaving paid work, working part-time and looking for a new job. This effect is stronger for people who had a physically demanding job. This suggests the importance of working conditions in shaping older workers’ employment behaviours.

